# Microtubule Formation and Activities of Antioxidative Enzymes in PC12 Cells Exposed to Phosphatidylcholine Hydroperoxides

**DOI:** 10.3390/ijms131215510

**Published:** 2012-11-22

**Authors:** Yukako Yamanaka, Shumi Yoshida-Yamamoto, Hiroshi Doi

**Affiliations:** Department of Food Science and Nutrition, Mukogawa Women’s University, Nishinomiya, Hyogo 663-8558, Japan; E-Mails: syoshida@mukogawa-u.ac.jp (S.Y.-Y.); hiroshid@mukogawa-u.ac.jp (H.D.)

**Keywords:** neurodegeneration, oxidative stress, phosphatidylcholine hydroperoxide, lipid, antioxidative enzyme

## Abstract

Aging increases free radical generation and lipid oxidation and, thereby, mediates neurodegenerative diseases. As the brain is rich in lipids (polyunsaturated fatty acids), the antioxidative system plays an important role in protecting brain tissues from oxidative injury. The changes in microtubule formation and antioxidative enzyme activities have been investigated in rat pheochromocytoma PC12 cells exposed to various concentrations of phosphatidylcholine hydroperoxides (PCOOH). We measured three typical antioxidative enzymes, superoxide dismutase (SOD), glutathione peroxidase (GPx), and catalase (CAT). The microtubule assembly system was dependent on the antioxidative enzyme system in cells exposed to oxidative stress. The activities of the three enzymes increased in a PCOOH exposure-dependent manner. In particular, the changes in the activity as a result of PCOOH exposure were similar in the three antioxidative enzymes. This is the first report indicating the compatibility between the tubulin-microtubule and antioxidative enzyme systems in cells that deteriorate as a result of phospholipid hydroperoxide administration from an exterior source. The descending order of sensitivity of the three enzymes to PCOOH is also discussed.

## 1. Introduction

Microtubules are important components of several subcellular structures, including the mitotic apparatus, cilia, flagella, and neurons, and play many roles in cellular processes, such as cell division, cell motility, and morphogenesis, which are required for brain function. A cellular shrinkage and a decrease in cell number have been observed. Matsuyama *et al*. [1] have hypothesized that impairment of the microtubule system is important to explain the pathogenesis of Alzheimer’s disease (AD). In addition, it has been reported that the AD brain has decreased synapse density [2]. Iqbal *et al*. [3] have reported on the inhibition of microtubule formation due to the kubextensive phosphorylation of the tau protein, which leads to microtubule degeneration in AD. We have reported on the inhibition of microtubule formation by phosphatidylcholine hydroperoxides (PCOOH) from soybean phosphatidylcholine (PC) [4]. The PCOOH used here was obtained by photooxidation of soybean PC. The high performance liquid chromatograms (HPLC) of PCOOH and PC are as found in the references. Soybean PC is mainly composed of palmitic acid (16:0) and linoleic acid (18:2). This PC is used in place of human cell PC. We have also presented some evidence of nerve cell dysfunction caused by microtubule disorder [5]. A remarkable increase of lipid peroxides in the brain with dementia has also been reported [6–8]. Using a neural cell line, PC12, it has been demonstrated that the damage of microtubules by beta-amyloid causes morphological abnormalities in the cell [9]. It is well known that oxidative stress plays a crucial role in neurodegenerative diseases, such as AD [10,11]. Organisms have developed mechanisms for their protection against oxidative stress. Intercellular ROS production and propagation are controlled by highly complex and integrated antioxidant systems [12]. The antioxidant enzymes in our body play an important part in aging. Enzymes, such as superoxide dismutase (SOD), glutathione peroxidase (GPx), and catalase (CAT), make up the most important endogenous antioxidant system. There are some papers investigating alterations in the antioxidant enzyme activities and an oxidative damage in different tissues of diabetic animals [13,14]. We have focused the compatibility between the tubulin-microtubule and antioxidative enzyme systems in cells exposed to oxidative damage. Lipid peroxidation products accumulate in the brains of people with AD, whereas the enzymatic antioxidant system exists in the brain. We found, too, that even very low concentrations of PCOOH are sufficient to interfere with a component of microtubule tubulin; therefore, microtubule formation and the interaction mechanism are hydrophobic [15]. As mentioned before, the PCOOH used in this study was obtained by photooxidation of soybean PC. The composition of soybean PC is similar to that of, for example, human platelet PC, which is composed mainly of palmitic acid (16:0) and eicosapentaenoic acid [16]. Although soybean PC is used instead of PC from human cells, behavior of PCOOH from soybean and human cells are thought to be similar. As the interaction between PCOOH and tubulin is hydrophobic in nature [4], we can expect to observe similar damage in cells exposed to human platelet PCOOH and cells exposed to soybean PCOOH. Considering that neurodegenerative diseases, such as AD, are induced by a microtubule disorder based on oxidative stress, endogenous enzymatic antioxidant systems are very important from the viewpoint of protection from such diseases. There are no data about the relationship between microtubule formation and the enzymatic antioxidant system in cells exposed to oxidative stress, whereas data on the disproportion between free radical levels and the enzymatic antioxidant system in cerebral regions of the aging rat have been reported [17]. It is very important to examine the comparative data from the viewpoint of the use of another nonenzymatic antioxidant system for the prevention of and protection from oxidative stress. We proposed in earlier research [18] that our experimental system using PCOOH and PC12 cells may be a good model of brain cells in the early stages of neurodegenerative diseases and showed microtubule formation in the cell in the presence of PCOOH. In addition, cell viability was significantly decreased in PC12 cells treated with PCOOH. Also our results with immunofluorescence microscopy using an anti-α-tubulin antibody revealed that PCOOH degrades tubulin and disrupts microtubules. These results led us to consider that the precise target of PCOOH in the neuron might be tubulin. The aim of this study is to clarify the effect of the disintegration of the antioxidant enzymatic system on microtubule formation in PC12 cells exposed to PCOOH and gather information on a precise target of PCOOH. Achieving this goal is dependent on assessing whether the increased PCOOH, as oxidative stress, causes the dysfunction of the antioxidant enzymatic systems of SOD, GPx, and CAT and the microtubule disorder by measuring guanosine triphosphatase (GTPase) activity as a putative index for microtubule formation.

## 2. Results

### 2.1. Inhibition of Microtubule Formation by PCOOH

Neurites consist mainly of microtubules, whose function is significantly based on the ability of tubulin to polymerize and depolymerize. Guanosine-5′-triphosphatase (GTPase) activity is an indicator of microtubule formation and, therefore, provides the degree of microtubule assembly [19–21].

**Figure 1:**
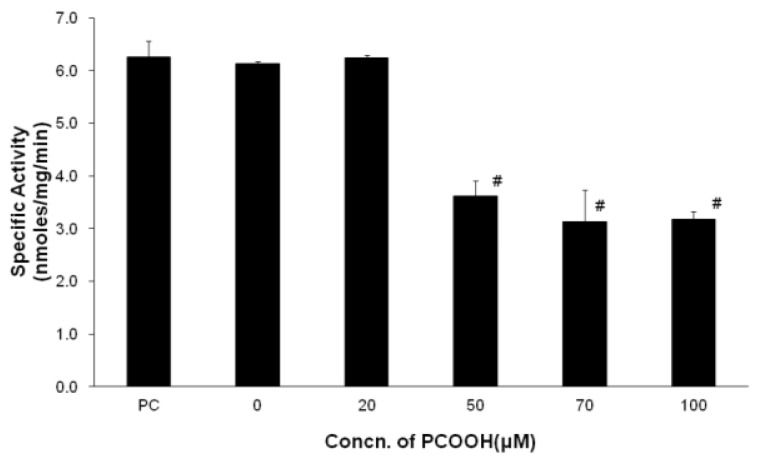
Guanosine-5′-triphosphatase (GTPase) activity of differentiated PC12 cells exposed to phosphatidylcholine (PC) or 0, 20, 50, 70 and 100 μmol/L phosphatidylcholine hydroperoxides (PCOOH). Differentiated PC12 cells were incubated in a medium containing PC or PCOOH for 24 h. The GTPase activity in the cell lysates was assayed by measuring the increase of guanosine diphosphate GDP by high performance liquid chromatography (HPLC) described in the previous report [15]. The data represent the means ± SD. ^#^*p* < 0.01 compared with the control value.

We measured the GTPase activity of cell extracts derived from differentiated PC12 cells exposed to different concentrations of PCOOH (Figure 1). The GTPase activity of cells exposed to 20 μM PCOOH hardly changed compared with that of cells incubated in the absence of PCOOH. The specific activity of GTPase of differentiated PC12 cells was decreased by half by exposure to 50 μM PCOOH. On the other hand, the activity was almost the same for 50 μM PCOOH exposure and 100 μM PCOOH exposure, while the PCOOH concentration increased from 50 μM to 100 μM. The microtubule formation pattern of differentiated PC12 cells exposed to PCOOH was a reverse sigmoid according to the PCOOH concentration, as shown in Figure 1. Microtubule formation was inhibited by PCOOH [4]. Native phosphatidylcholine (PC) and 20 μM PCOOH exposures did not seem to affect microtubule formation due to the endogenous antioxidative system, and excess PCOOH was not eliminated by the system.

### 2.2. Effects of PCOOH on SOD Activities

The enzyme SOD exists in the cytoplasm of all cells in great abundance. SOD catalyzes the breakdown of the superoxide anion (O_2_•^−^) by transforming the superoxide anion into a product, such as hydrogen peroxide. SOD eliminates the superoxide, which creates the hydroxyl radical in the presence of iron and cupper ion. Hydrogen peroxide is metabolized to water by GPx. SOD is mainly located in neurons. These findings mean that SOD provides the first line of defense against oxygen toxicity in neurons.

**Figure 2:**
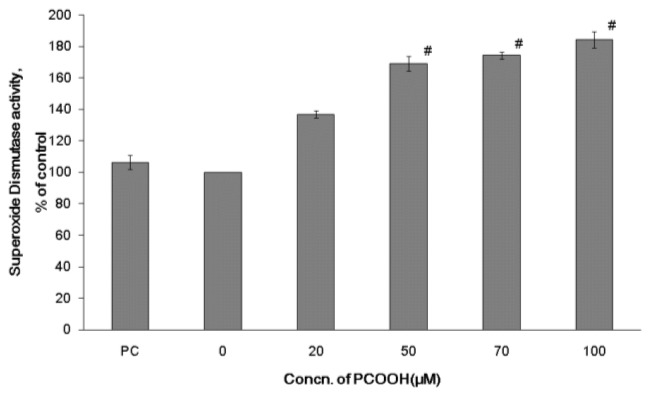
Superoxide dismutase (SOD) activity of differentiated PC12 cells exposed to PC or 0–100 μmol/L PCOOH. After incubation in the presence of PC or PCOOH, the original medium was removed, and cells were lysed with a Nonidet P-40 lysis buffer. The level of SOD activity in the cell lysates was measured using the SOD Assay Kit-WST (Dojindo Molecular Technologies, Inc., Gaithersburg, MD, USA) according to the manufacturer’s instructions. Data are experiments as percent of the control; the mean value was obtained from six experiments. The data represent the means ± SD. ^#^*p* < 0.01 compared with the control value.

We measured the SOD activity of differentiated PC12 cells exposed to PCOOH at various concentrations for 24 h using a SOD measurement kit. As shown in Figure 2, cells exposed to 50 μM PCOOH showed highly increased SOD activity relative to those exposed to 20 μM PCOOH. In cases of cells exposed to 50, 70, and 100 μM PCOOH, the SOD activities were significantly higher than those of cells exposed to no or 20 μM PCOOH. The SOD activity of cells exposed to 50, 70, and 100 μM PCOOH increased 1.69-, 1.74-, and 1.84-fold, respectively, relative to that of those exposed to no PCOOH. The effect of native PC was not significant.

The SOD activity of the PC12 cell increased depending on the PCOOH concentration in the presence of over 50 μM PCOOH. The activity seemed to increase in order to eliminate O_2_^−^ in the cells induced by PCOOH. The facts obtained in this experiment support the idea that SOD activity can be induced by peroxidative stress [22].

### 2.3. Effects of PCOOH on GPx Activities

GPx reduces hydrogen peroxide generated from the dismutation of O_2_•^−^ by SOD and exchanges hydroperoxides from membrane lipid and free fatty acid into corresponding alcohols within a cell. Therefore, GPx protects intracellular ingredients from oxidative denaturation and plays a role in stabilizing each organelle membrane. GPx is an essential factor for the viability of aerobic organisms.

**Figure 3:**
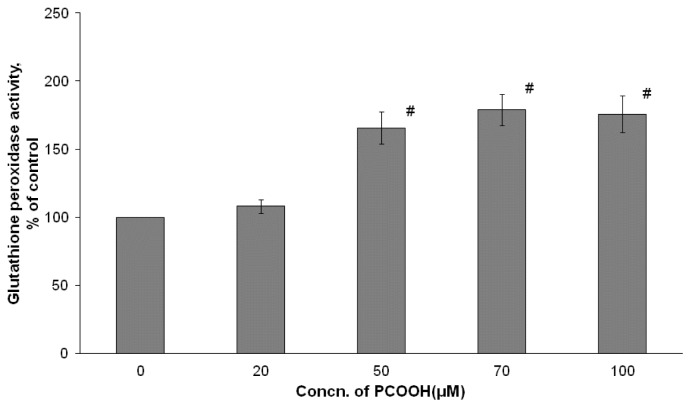
Glutathione peroxidase (GPx) activity of differentiated PC12 cells exposed to 0–100 μmol/L PCOOH. After incubation in the presence of PCOOH, the cells were washed with ice-cold phosphate buffered saline (PBS), and homogenized in a lysis solution containing 5 mM EDTA/0.01% digitonin/0.25% sodium chlorate. After centrifugation, the enzymes in the supernatants were assayed [23]. The activity of GPx was measured with the Bioxytech GPx-340 kit. Data are experiments as percent of the control; the mean value was obtained from six experiments. The data represent the means ± SD. ^#^*p* < 0.01 compared with the control value.

As shown in Figure 3, the GPx activity increased by 8.2% of the control value of the PC12 cell with 20 μM PCOOH exposure. The GPx activity significantly increased by 65.7%, 79.1%, and 75.8%, respectivey, with exposure to PCOOH at concentrations of 50, 70, and 100 μM compared with the value with no PCOOH. There is a slight difference between the GPx activity with no exposure to PCOOH and that with exposure to 20 μM. However, the GPx activity went up rapidly when changing the PCOOH concentration from 20 to 50 μM. In the case of cells incubated under the condition of 50–100 μM PCOOH exposure, the GPx activity was almost the same. The capability to eliminate hydrogen peroxide from the cells exposed to 50 μM PCOOH was higher than that from cells exposed to 20 μM PCOOH. The GPx activity pattern with the PCOOH concentration was sigmoidal, as shown in Figure 3.

### 2.4. Effects of PCOOH on CAT Activities

CAT is one of the most abundant enzymes in nature and is widely distributed throughout tissues in the animal and plant kingdom. At the cellular level, it is located in the cytosol. This enzyme is involved in the destruction of hydrogen peroxide generated during cellular metabolism.

**Figure 4:**
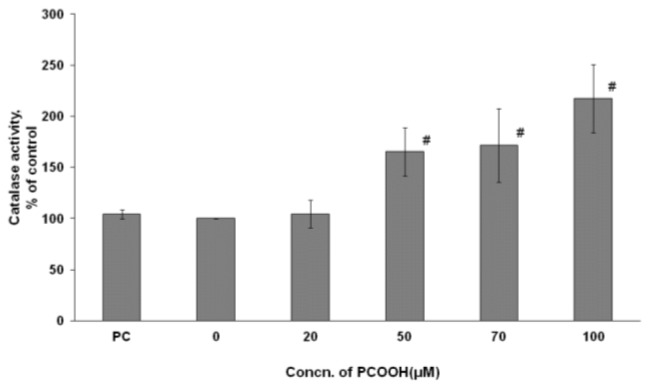
Catalse (CAT) activity of PC12 cells exposed to PC or 0–100 μmol/L PCOOH. After incubation in the presence of PC or PCOOH, a phosphate buffer, methanol, H_2_O_2_, and distilled water were mixed. Then, the mixed solution, sample protein, and a phosphate buffer were incubated for 20 min, and Purpald was added. The mixture was incubated for 20 min. Finally, potassium periodate was added, and the absorbance was measured at 550 nm. Data are experiments as percent of the control; the mean value was obtained from six experiments. The data represent the means ± SD. ^#^*p* < 0.01 compared with the control value.

Figure 4 shows that the intracellular CAT activity became higher with 50–100 μM PCOOH exposure than with no PCOOH exposure. While exposures to native PC and 20 μM PCOOH had no effect on the activity, exposures to 50, 70, and 100 μM PCOOH significantly increased the CAT activity of differentiated PC12 cells, and the increase percentiles were 65.3%, 71.5%, and 117.3%, respectively. There was no significant difference between the CAT activity of cells exposed to 50 μM PCOOH and that of those exposed to 70 μM PCOOH (Figure 4). The increase of CAT activity by exposure to PCOOH appeared to be composed of two steps under experimental conditions. The first step was observed between the exposure to 20 μM PCOOH and that to 50 μM PCOOH, and the next, between the exposure to 70 μM PCOOH and that to 100 μM PCOOH. The tendency of the activity changes observed in CAT was very similar to that observed in GPx. The activity of CAT in the cells exposed to PCOOH became extremely large when raising the PCOOH concentration from 20 μM to 50 μM in the medium. The results indicate that CAT activity is induced by an excess amount of substrate.

## 3. Discussion

To understand the correlation between neurodegenerative diseases and microtubule disorders, we have been studying the effects of lipid hydroperoxides on microtubule formation [5]. In an earlier paper, we showed that PCOOH inhibited neurite outgrowth through microtubule disorders in PC12 cells [18]. Therefore, it is biologically interesting to elucidate the behavior of endogenous antioxidative systems under oxidative stress in neuronal cells. In addition, reports on the tubulin-microtubule system of neurodegenerative diseases are sparse. This is the first report indicating the association between the tubulin-microtubule system and enzymatic antioxidative systems in cells under oxidative stress. Furthermore, the most sensitive enzyme among the antioxidative enzymes is suggested to be SOD by specifying the enzyme that responded to the lowest concentration of PCOOH (Figure 2).

It is widely accepted that oxidative stress is significantly related to the development of disease and that this relationship increases in connection with many lifestyle-related diseases and aging [22,24–26]. There have been numerous studies on reactive oxygen species and aging since the relationship between them was initially proposed by Harman [27–31]. Furthermore, it is clear that reactive oxygen species, such as hydrogen peroxide, hydroperoxides, and free radicals, oxidize directly with biomolecules and organelles and also act as signal messengers in intracellular communication [32].

Considering the effects of oxidative stress, it is important to examine the balance of antioxidative defenses, repair of oxidative damage, and generation of oxidants as well as products due to reactive oxygen species [33]. Humans have various defense networks that include anti-oxidization elements and enzymatic antioxidative systems that protect against oxidative stress. Antioxidant enzymes are important protective mechanisms against oxidative stress [34–36].

In this study, we examined the change of enzymatic antioxidative systems in response to PCOOH as an oxidative stressor in differentiated PC12 cells. The PCOOH concentrations to which cells were exposed were 0, 20, 50, 70, and 100 μM in the culture medium in order to clarify the effects of PCOOH on both antioxidant enzymatic activity and microtubule formation, while they were 0, 50, and 100 μM in our previous report [18]. The GTPase activity of differentiated PC12 cells incubated under a culture condition of 20 μM PCOOH was slightly lower than that under a condition of no PCOOH (Figure 1). The GTPase activities of differentiated PC12 cells decreased much more significantly with exposures to over 50 μM PCOOH than did those of cells exposed to 20 μM PCOOH. This is the first report in which the effect of phosphatidylcholine hydroperoxides on microtubule formation has been observed in detail.

In parallel with the results of GTPase activity, a microtubule disorder was not observed when the PCOOH concentration to which differentiated PC12 cells were exposed was under 20 μM. On the other hand, raising the PCOOH concentration to 50 μM induced a decrease of microtubule formation (Figure 1). The concentration of oxidants in cells increases with age. The results shown in Figure 1 indicate that a microtubule disorder might appear in an aged neuronal cell. In other words, the endogenous antioxidant system was insufficient to maintain normal microtubule formation. This indicates that another exogenous antioxidant system is necessary to protect microtubule formation.

The antioxidant enzyme system in the cell exposed to PCOOH worked to eliminate hydrogen peroxide, phospholipid hydroperoxide, and superoxide. The GTPase activity of cells exposed to 20 μM PCOOH was not different from that of cells exposed to no PCOOH.

Even when differentiated PC 12 cells were exposed to 20 μM PCOOH, SOD was activated, while GPx was activated with exposure to 50 μM PCOOH (Figures 2 and 3). In the case of CAT, the exposure to 50 μM PCOOH resulted in a slight increase, and that to 100 μM PCOOH produced a large increase (Figure 4). These results indicate that antioxidant enzymes were activated in the presence of PCOOH by SOD, GPx, and CAT, in that order. This order also indicates the sensitivity among the examined enzymes.

In the case of cells incubated with over 50 μM PCOOH, recovery of GTPase activity (tubulin-microtubule system) was not observed, while the three antioxidative enzymes caused its activity to increase. This result indicates the limitations of the ability of an antioxidative enzyme to remove oxidative stress induced by hydrogen peroxide, lipid hydroperoxides, and superoxide.

The changes in the GTPase activity and the antioxidative enzyme induced by exposure of cells to PCOOH are associated. This means that these enzyme activities are associated with each other and depend on the remaining amount of reactive oxygen species (Figures 1–4).

When differentiated PC12 cells were exposed to PCOOH, the enzymatic antioxidative systems did not change markedly under 20 μM, and a significant surge in the activities was observed over 50 μM. The same patterns were observed in the assay using three enzymes.

The effect of PCOOH exposure on microtubule formation and antioxidative enzymes was compatible. For example, the pattern indicated in Figure 3 is the complete reverse of that in Figure 1, indicating that the effect of PCOOH on microtubule formation was associated with the induction of enzymatic antioxidative systems by lipid hydroperoxides. This means that the precise target would be PCOOH in aged cells.

As described in a previous paper, this experimental system might be a good model for clarifying the aging mechanism by lipid hydroperoxides. The result obtained in this study supports a proposal in our previous study that one of the targets of PCOOH is tubulin. In a typical protective system, reactive oxygen species derived from PCOOH would be eliminated and cells would function satisfactorily. On the other hand, even if antioxidative enzyme activities arise in response to an excess concentration of PCOOH, the reactive oxygen species that is cleaved completely would remain in a cell. The concentration of phosphatidylcholine hydroperoxides used in this paper was appropriately compared with that present in living cells. Although phosphatidylcholine hydroperoxides resulted in the increase of the three main antioxidative enzyme activities in this study, GTPase activity decreased. This fact indicates that microtubule formation was not sufficient. These phenomena would be possible in living cells and would lead to aging and neurodegenerative diseases. Antioxidative enzymes were effective in eliminating reactive oxygen species, but the results of GTPase activity obtained in this study suggest that the enzymes were not effective in this regard. This leads to the deterioration of cell function by reactive oxygen species. It is assumed that an enzymatic antioxidative system would not work in cases of aging and neurodegenerative diseases. Our findings in this study identifying the compatibility of GTPase activity and antioxidative enzyme activity are very important for the understanding of the function of a brain with dementia.

In this study, we examine the compatibility of microtubule formation and antioxidative enzyme activity. The use of exogenous antioxidants should be considered for the removal of superoxide anions based on their sensitivity to exposure to oxidative stress. It is important to observe the behavior of microtubule formation in the presence of various antioxidants. Experiments using antioxidants are now in progress.

## 4. Experimental Section

### 4.1. Materials

PC12 cells were purchased from Dainippon Sumitomo Pharma Co., Ltd. (Osaka, Japan). Fetal bovine serum (FBS), horse serum (HS), and penicillin/streptomycin were obtained from ICN Biomedicals, Inc. (Aurora, OH, USA). Nerve growth factor-7S (NGF-7S) was from Sigma Chemical Co. (St. Louis, MO, USA). The RPMI-1640 medium was obtained from GIBCO Invitrogen Corporation (New York, NY, USA). The starting material used to prepare phosphatidylcholine hydroperoxide (PCOOH) was Lipoid S-100, a soybean phosphatidylcholine (PC), which is essentially pure PC, obtained from Nisshin Oilio Group, Ltd. (Tokyo, Japan). The method for preparing PCOOH has been described previously [15]. For example, 20 μM hydroperoxide corresponded to 0.038mg/mL of PCOOH. The concentration of PCOOH used here was several times higher than that used for *in vivo* measurements [37–39]. To assess the causes of cell damage, we performed a series of experiments using normal phospholipids as substitutes for PCOOH at the same concentrations. Chemicals used were of reagent grade.

### 4.2. Cell Culture

The PC12 cell line was derived from transplantable rat adrenal pheochromocytoma [40]. Differentiated cells were used in every experiment discussed in this paper. Differentiated cells were more sensitive to phosphatidylcholine hydroperoxides than undifferentiated cells described in our previous paper [18], which suggested that microtubules were an important target of phosphatidylcholine hydroperoxides. PC12 cells were propagated in an RPMI-1640 medium containing 10% heat-inactivated HS, 5% heat-inactivated FBS, and 0.3% penicillin/streptomycin. Cells were cultured at 37 °C in humidified 95% air with 5% CO_2_. The cells differentiated into responding NGF by induction of the neuronal phenotype. For differentiation, the undifferentiated cells were seeded on dishes or plates. After 24 h of incubation, the culture medium was replaced with a fresh medium containing 0.5% HS and 100 ng/mL NGF-7S. All cells were cultured in collagen-coated culture dishes, flasks, or plates.

### 4.3. Exposure of PC12 Cells to PCOOH

Differentiated PC12 cells were exposed to PCOOH by replacing the RPMI medium containing 1% HS and 100 ng/mL NGF-7S with PCOOH at a concentration between 0 and 100 μM, and cells were incubated for 24 h. The concentration of PCOOH was determined by its extinction coefficient at 233 nm, as described before [4,18]. Before the enzyme assay experiments, cells were washed with the RPMI-1640 medium and gathered by centrifugation.

### 4.4. Assay of GTPase Activity

Differentiated PC12 cells were incubated in a medium containing PC or 0, 20, 50, 70, and 100 μM PCOOH for 24 h. Treated cells were gathered by centrifugation and disrupted using a mini-beater (Biospace Products, Bartlesville, OK, USA) in a 10 mM sodium phosphate buffer, pH 7.0. After brief centrifugation, the cell lysate was obtained. The GTPase assay was performed by adding an aliquot of supernatant to a 10 mM sodium phosphate buffer, pH 7.0, containing 0.1 mM GTP, 3.4 M glycerol, and 10 mM MgCl_2_. GDP, the reaction product, was quantified by the HPLC system according to a previous report [15]. The protein quantity was determined with the Bradford protein assay (Bio-Rad Laboratories Inc., Hercules, CA, USA).

### 4.5. Antioxidant Enzymes

#### 4.5.1. Determination of SOD Activity

The level of SOD activity in differentiated PC12 cells was measured using the SOD Assay Kit-WST (Dojindo Molecular Technologies Inc., Gaithersburg, MD, USA) according to a protocol described previously [41]. After incubation of the cells with the experimental reagents for 24 h, the original medium was removed from the 96-well plate, and the cells were lysed with the Nonidet P-40 lysis buffer (1%NP-40, 50 mmol/L Tris-HCl (pH 7.5), 0.05 mM ethylenediamine tetra-acetate) for 20 min at 4 °C. The lysates were centrifuged at 300× *g* for 10 min, and 20 μL of the supernatant was used for determination of the SOD activity according to the manufacturer’s instructions. The value for each treatment group was converted to the percentage control.

#### 4.5.2. Measurement of GPx Activities

Differentiated PC12 cells were centrifuged, washed with ice-cold PBS, and homogenized in 500 μL of a lysis solution containing 5 mM EDTA/0.01% digitonin/0.25% sodium chlorate. After centrifugation at 10,000× *g* for 30 min at 4 °C, the enzymes in the supernatants were assayed [23]. The activity of GPx was measured with the Bioxytech GPx-340 kit (OXIS International, Portland, OR, USA).

#### 4.5.3. Determination of CAT Activity

The CAT activity was determined as previously described [42]. Briefly, a 0.25 M phosphate buffer, 12 M methanol, 44 mM H_2_O_2_, and distilled water were mixed. Then, the mixed solution, sample protein, and a phosphate buffer were incubated for 20 min, and Purpald (Sigma-Aldrich, St. Louis, MO, USA) (25 mM in 2 N potassium hydroxide) was added to formaldehyde made from the reaction as a coloring substrate; the mixture was incubated for 20 min. Finally, 65.2 mM of potassium periodate was added to stop the reaction completely, and the absorbance of the purple formaldehyde adduct was measured at 550 nm [43].

### 4.6. Statistical Analysis

Data are expressed as means and standard deviations. Differences in the mean values were assessed for significance by one-way analysis of variance (ANOVA). To compare undifferentiated and differentiated cells, the non-paired *t*-test was applied.

## 5. Conclusions

It is important to observe the behavior of microtubule formation as well as antioxidative enzyme activities of cells deteriorated by PCOOH because microtubules play have roles, such as cell division, cell motility, and morphogenesis, and are also required for brain function. The compatibility between the tubulin-microtubule system and the antioxidative enzyme system in cells deteriorated by PCOOH is clearly demonstrated in this paper. The tubulin-microtubule system of differentiated PC12 cells decreased significantly with exposure to PCOOH dependent on concentration, while the three antioxidative enzymes increased in activity. This result indicates the limitations of the ability of an antioxidative enzyme to remove oxidative stress induced by hydrogen peroxide, lipid hydroperoxides, and superoxide. Our findings from the compatibility of the tubulin-microtubule system and antioxidative enzyme activity are very important for the understanding of brain function in individuals with dementia.

The microtubules should be sufficiently formed for proper function in the cells. Based on the facts of the decrease of microtubule formation and the increase of antioxidative enzyme activities in the cells deteriorated by PCOOH, cells need additional antioxidative activities for normal viability. Therefore, the use of exogenous antioxidants should be considered.
